# Contribution of biofilm formation genetic locus, *pgaABCD*, to antibiotic resistance development in gut microbiome

**DOI:** 10.1080/19490976.2020.1842992

**Published:** 2020-11-16

**Authors:** Dachuan Lin, Kaichao Chen, Jiubiao Guo, Lianwei Ye, Ruichao Li, Edward Wai Chi Chan, Sheng Chen

**Affiliations:** a Guangdong Provincial Key Laboratory of Regional Immunity and Disease, Department of Pathology Biology, School of Medicine, Shenzhen University, Shenzhen, China; b State Key Laboratory of Chemical Biology and Drug Discovery, Department of Applied Biology and Chemical Technology, The Hong Kong Polytechnic University, Kowloon, Hong Kong; c Department of Infectious Diseases and Public Health, Jockey Club College of Veterinary Medicine and Life Sciences, City University of Hong Kong, Kowloon, Hong Kong; d Jiangsu Co-Innovation Center for Prevention and Control of Important Animal Infectious Diseases and Zoonoses, College of Veterinary Medicine, Yangzhou University, Yangzhou, People’s Republic of China

**Keywords:** Microbiome, *Escherichia coli*, sub-species diversity, antimicrobial resistance progenitor cells

## Abstract

The human gut microbiome is the presumed site in which the emergence and evolution of antibiotic-resistant organisms constantly take place. To delineate the genetic basis of resistance formation in gut microbiome strains, we investigated the changes in the subpopulation structure of *Escherichia coli* in rat intestine before and after antimicrobial treatment. We observed that antibiotic treatment was selected for an originally minor subpopulation *E. coli* carrying the biofilm-forming genetic locus *pgaABCD* and the toxin-antitoxin system *HipAB*. Such strains possessed dramatically enhanced ability to withstand the detrimental effects of antibiotics, becoming a dominant subspecies upon antibiotic treatment and eventually evolving into resistant mutants. In contrast, *E. coli* strains that did not carry *pgaABCD* and *HipAB* were eradicated upon antibiotic treatment. Our findings, therefore, suggested that genes encoding biofilm-forming ability played an important role in conferring specific gut *E. coli* strains the ability to evolve into resistant strains upon a prolonged antibiotic treatment, and that such strains may therefore be considered bacterial antibiotic resistance progenitor cells in the gut microbiome.

## Introduction

Clinical and public health problems due to multidrug-resistant bacterial infections have worsened in recent years due to the continual emergence of bacterial strains harboring novel and transmissible antibiotic resistance genes.^1-3^ Drug-resistant infections are already estimated to cause 700000 annual deaths globally and an estimate of 5·7 million deaths annually are the result of a lack of access to antibiotics.^3^ An extremely large number and a wide variety of microbes are known to inhabit the gastrointestinal tract of mammals, with 60% of the organisms in the human gut microbiome surviving for as long as 5 years on average.^4,5^ In recent years, however, human and animal feces are implicated to be the major source of microbial pathogens that cause antibiotic-resistant hospital and food-borne infections, prompting us to hypothesize that the human and animal gut microbiome is the primary biological system in which novel antibiotic-resistant strains are generated, presumably *via* evolution events driven by selection pressure imposed by antibiotics from time to time, and that such events lead to long-lasting or even permanent change in the population structure of the gut microbiome in both animals and human. The gut microbiome of both human and animals fits all the following criteria as an ideal breeding ground for new resistant strains: (i) bacteria are periodically exposed to antibiotics, resulting in induction of mutational changes and selection of existing resistant strains, (ii) resistant organisms can transfer resistance-encoding genetic materials to other antibiotic-susceptible organisms, producing new resistant strains, (iii) resistant bacterial sub-populations have the chance to proliferate and become dominant, (iv) resistant organisms may be released to the environment, and (v) resistant organisms can readily cause opportunistic infections in human. However, the genetic basis underlying the emergence of phenotypically novel resistant organisms from the gut microbiome remains ill-defined.

To probe the evolutionary events inducible by antibiotics, we analyzed the changes in the population structure of the gut microbiome in rats which have never been exposed to antibiotics, and found that resistant strains were consistently derived from specific subpopulation progenitor strains which exhibited a wide range of genetic alterations when compared to the majority of the gut microbiome population. We identified a specific subpopulation of gut microbiome *E. coli* strains that harbor a range of unique genetic elements, including the known biofilm genes *pgaABCD*, that encode biofilm-forming functions.^6^ Such strains, therefore, possess dramatically enhanced ability to withstand the detrimental effects of antibiotics and evolve into resistant mutants during the process of antimicrobial treatment. We further showed that the majority of antibiotic-resistant clinical isolates exhibited genetic characteristics of the gut microbiome subpopulation strains identified in this work, confirming that resistant organisms predominantly evolved from such progenitor strains. Possibly termed resistance progenitor cells, the discovery of such strains has important implications in the development of novel strategies to prevent the emergence and evolution of resistant organisms in the intestine, and hence drastically reduce the rate of drug-resistant opportunistic infections, especially in the hospital environment.

## Results

### Subspecies diversity of GI tract microbiome facilitates adaption to antibiotic selection pressure

**Figure 1.**
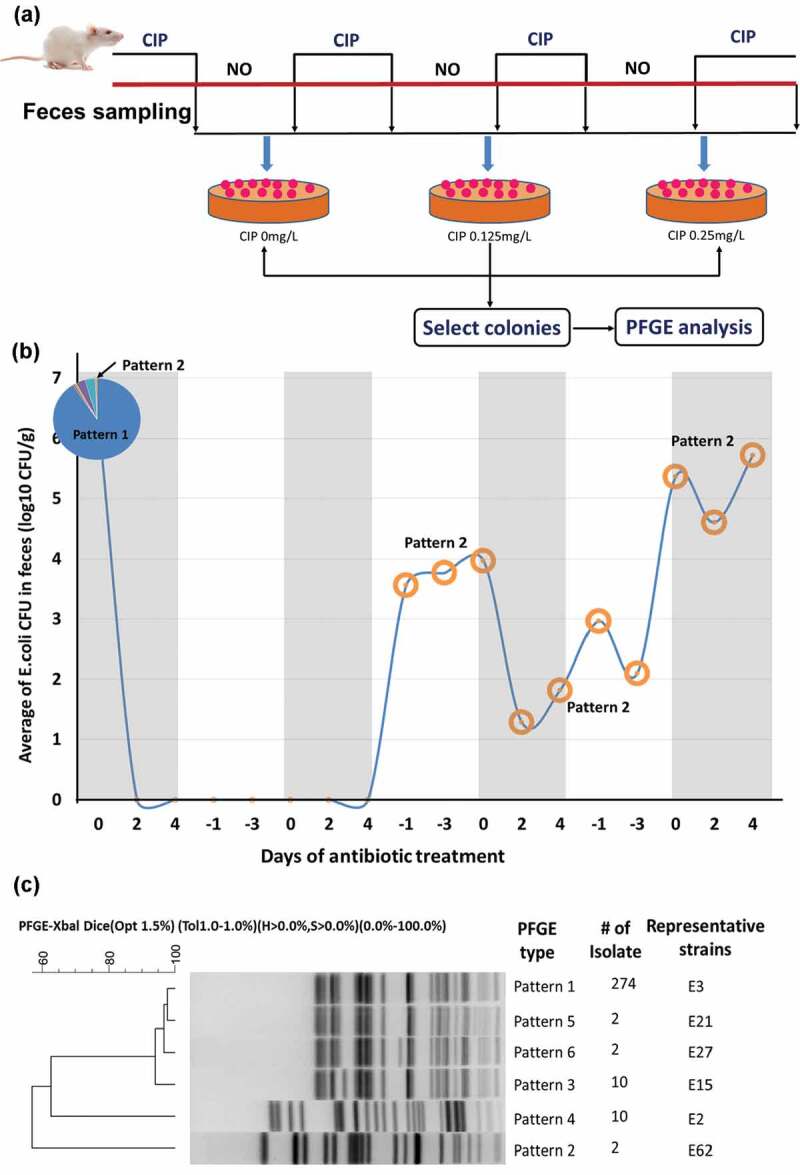
**Dynamic changes in the average population size and genetic types of *E. coli* in fecal samples of three rats during consecutive cycles of ciprofloxacin challenge and withdrawal**. (a) Illustration of the experimental procedure. (b) Population size in log _10_ CFU/g. The numbers 0, 2, 4, −1, −3 in X-axis respectively represent the day on which treatment was initiated, 2 days of treatment, 4 days of treatment, 1 day after withdrawal of treatment, and 3 days after withdrawal of treatment. The pie chart represents the portion of strains exhibiting different PFGE patterns at the beginning of the first treatment round. The *E. coli* population in fecal samples was almost completely eradicated during the first two rounds of antibiotic challenge but began to expand again during the second antibiotic-withdrawal period. A minority strain exhibiting pattern 2 in the original population became the predominant organism since then. (c) Dendrogram showing results of cluster analysis of the Xbal-PFGE profile of *E. coli* strains collected before antibiotic treatment. The degree of similarity of the PFGE patterns of the test strains was calculated by BioNumerics 5.1 (Applied Math, Kortrijk, Belgium), using the conventional criteria, with Dice coefficient being at 1.5% optimization (Opt) and 1% position tolerance (Tol). In this comparison analysis, H (minimum height) and S (minimum surface) were set at 0%, with coverage of 0.0%–100% for the entire length. Three major patterns of the test isolates were observable. Remarkably, a total of 44 strains collected after treatment were found to belong to pattern 2

In a previous study, we demonstrated that ciprofloxacin-resistant *E. coli* strains consistently emerged in the gastrointestinal (GI) tract of the rat upon prolonged treatment with various dosages of ciprofloxacin.^7^ Such finding prompted us to initiate the current study and investigate the cellular mechanisms that govern the response of gut microflora to antibiotic treatment, using rat as the test animal and *E. coli* as a model gut microbiome strain. Consistent with the results of this previous study, we found that upon treatment of rats with a subtherapeutic dose (1 mg/kg body weight) of ciprofloxacin, *E. coli* in fecal samples completely disappeared after two rounds of antibiotic exposure. During the second antibiotic-withdrawal period, however, *E. coli* reemerged and the population size increased sharply, shrinking slightly during the third treatment round but increased to the original population size during the fourth episode of antibiotic exposure (Figure 1b). One rat in this treatment group was selected for further study. A total of 300 *E. coli* isolates collected from the rat prior to antibiotic treatment and each of 40 *E. coli* isolates collected at different treatment time points was subjected to PFGE analysis, which helped differentiate these *E. coli* isolates into six groups (Figure 1c). *E. coli* isolates collected prior to antibiotic treatment were found to comprise six major patterns, with pattern 1 accounting for the vast majority of the test strains (Figure 1b). Surprisingly, all organisms collected during subsequent treatment and cessation experiments were found to belong to pattern 2, one of the minor patterns detected prior to antibiotic treatment, indicating that only one subspecies of *E. coli* in the gut microbiome could survive and constituted the entire *E. coli* population long after the selection pressure of antibiotic had diminished.

**Figure 2.**
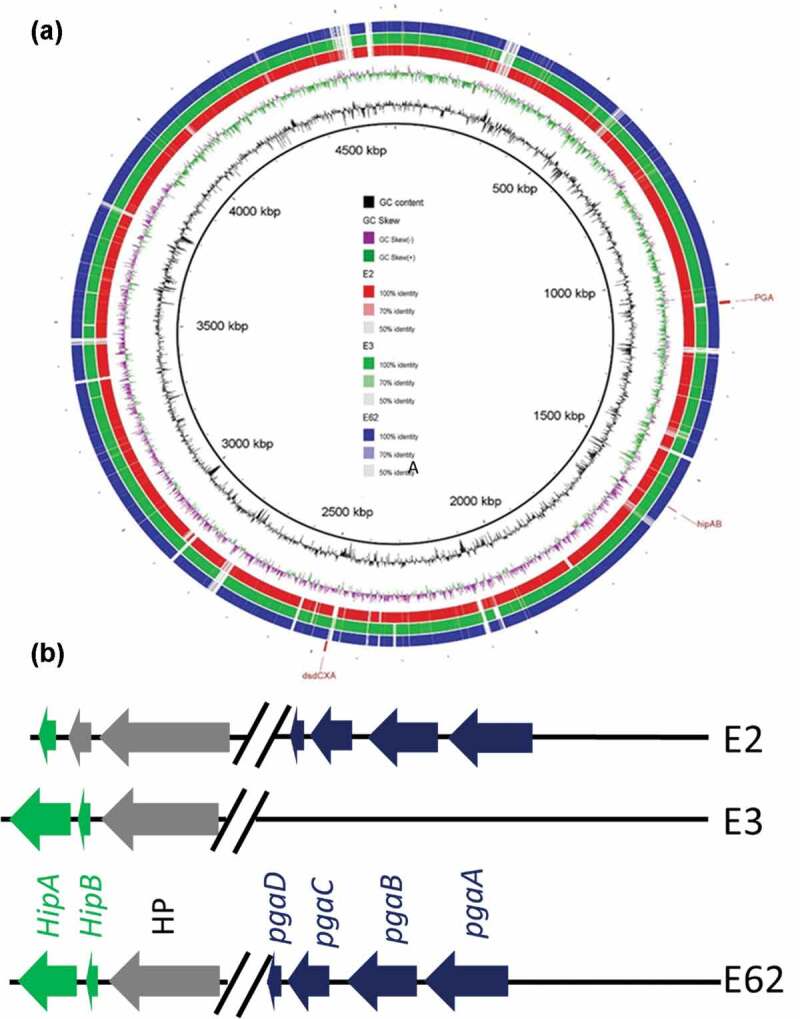
**PFGE and comparative genomic analysis of representative E. coli strains from gut microbiome before and after antibiotic treatment**. (a) Comparative genomic features of *E. coli* strains E2, E3 and E62. E21, E27 and E15 exhibited closer genetic arrangement as E3. The circular map depicts the degree of similarity among these three strains and the reference strain BW25113 as determined by BLASTN analysis. The figure was produced using BRIG.^8^ The major genetic difference between these three strains is shown in (b)

One each of the representative *E. coli* strain of pattern 1 ~ 6, named E3, E62, E15, E2, E21, and E27, respectively, were selected for further study. To confirm that pattern 2, E62, could survive better after ciprofloxacin treatment *in vitro*, the survival rate of strains E2, E3, and E62 during ciprofloxacin treatment was tested. Each of the three strains was incubated in LB supplemented with 0.5 μg/ml ciprofloxacin and the survival rate of E62 was found to be significantly higher than that of E2 and E3 (*p* < .005), with E2 in turn exhibiting a significantly higher survival rate than E3 (*p* < .005) (Figure 2a).

### *Genetic traits* pgaABD *and* hipAB *might contribute to the survival and subsequent evolution into resistant organisms of* E. coli *strain E62*

To dissect molecular mechanisms governing the dynamic change in the population structure of gut microbiome *E. coli* strains upon an antibiotic treatment, representative organisms of each PFGE pattern (Pattern 1 ~ 6) were randomly selected and subjected to whole genome sequencing, followed by the construction of a phylogenetic tree using SNP data and matching of the test strains against 52 representative strains in the NCBI genome database. E2 and E62 showed the closest genetic relationship, while others exhibited a close genetic relationship to each other, while distal to E2 and E62. E2, E6, and E62 were selected as representative strains and subjected to further analysis. E2 belonged to ST278, E3 belonged to unknown ST and E62 belonged to ST2521. SNP analysis between these three strains showed that 14337 SNPs were identified between E62 and E2, 17835 SNPs identified between E62 and E3, and 16671 identified between E2 and E3 (**Fig S1**).

**Figure 3.**
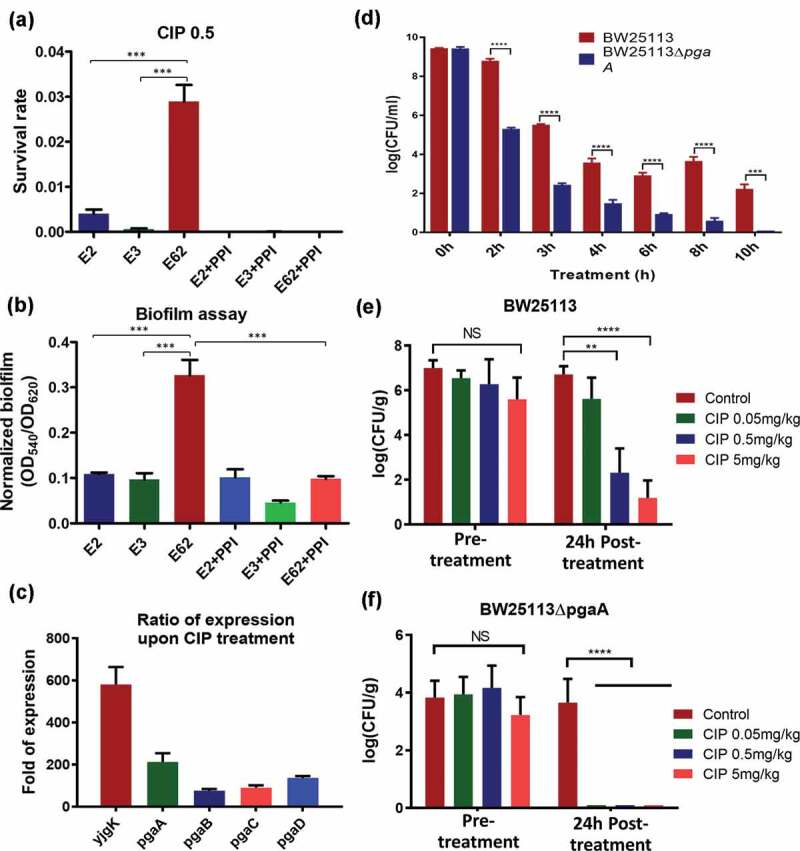
**Functional role of biofilm in conferring survival upon ciprofloxacin challenge**. (a) Survival rate of the test strains at CIP = .5 µg/ml in the presence and absence of esomeprazole at 32 μg/ml; (b) Biofilm formation potential of *E. coli* strains E2, E3 and E62 in the presence and absence of the Proton pump inhibitor (PPI) esomeprazole (32 μg/ml), which was reported to inhibit formation of biofilm in *E. coli*; (c) Changes in expression levels of putative biofilm genes in strain E62 with and without ciprofloxacin selection pressure. Fold changes were calculated after normalization with 16s rRNA. The *ygjK* gene, which encodes a glucosidase,^9^ is a biofilm modulator regulated by various toxins.^10^ (d) Effect of biofilm formation gene *pgaA* on E62 survival under ciprofloxacin selection pressure; (e) and (f) Survival of the wildtype and *pgaABC* knockout strain in rat GI tract before and after ciprofloxacin treatment. Data were analyzed using two-tailed unpaired student’s *t* test using Prism Graphpad 7.0

To depict the mechanisms underlying the better survival of E62 upon antibiotic treatment in animal GI tract, two representative *E. coli* strains, E3, which was the dominant *E. coli* strain detectable before antibiotic treatment, and E62, the dominant *E. coli* strain in gut microbiome after ciprofloxacin treatment, were subjected to analysis by PacBio SMRT and Illumina sequencing and the complete genome sequences of these strains were obtained through the hybrid assembly of Illumina reads and PacBio reads. Comparative genomics analysis of E3 and E62 identified more than 300 genetic differences between the genomes of these two strains, preventing us to identify unique genetic traits that contributed to E62 survival under ciprofloxacin pressure (Figure 3a**, Fig S2**). We then attempted to increase the numbers of genomes for comparison. We hypothesized that strains like E62 can survive better under ciprofloxacin pressure and would have a better chance to undergo mutational changes in target genes such as *gyrA*. We included 54 complete *E. coli* genome sequences in the NCBI genome database that harbored *gyrA* mutations and exhibited similar features of E62. The core genes of the dataset, defined as those shared by over 90% of the strains in the dataset, were shortlisted. Comparing these core genes with the genome of E3, eight genes that were absent from the E3 genome were identified. Among these eight genes, *gpr, rhaS, yjdJ, carD* and *dkgB* were known to be involved in nutrient utilization; *manZ* was mannose-specific PTS enzyme IID component; *yhbX* was an inner membrane putative hydrolase; and the last one was *pgaC*, which was uniquely present in strain E62 but not E3. The *pgaC* was in the *pgaABCD* cluster, which had been suggested to be involved in biofilm formation.^11–13^ We then tried to focus on this gene cluster for further analysis since biofilm formation has been demonstrated to be related to bacterial antimicrobial resistance.^14,15^ Other biofilm-related genes such as *hipAB* genes, which are one of the bacterial toxin/antitoxin (TA) systems in *E.coli*^16^ and known persistence markers, were also selected for comparison among E2, E3, and E62. Interestingly, E3 and E62 were found to harbor *hipAB*, whereas strains E2 and E62 were found to harbor the *pgaABCD* genes (Figure 2).

### *Ability to produce biofilm by* E. coli *strain E62 contributes to its survival under ciprofloxacin selective pressure*

We hypothesized that strain E62 exhibited an advantage to persist under ciprofloxacin pressure due to its ability to produce biofilm. Biofilm assays were then performed on these three strains, with results confirming that strain E62 exhibited a significantly higher level of production of biofilm than E2 and E3 (*p* < .005) (Figure 2b). Previous reports showed that the proton pump inhibitor (PPI) esomeprazole could inhibit biofilm formation and eradicate existing biofilm. Esomeprazole was then used to test whether biofilm formation was essential for the survival of strain E62 during ciprofloxacin treatment.^17^ In the presence of this compound, biofilm production in all three strains was found to be suppressed and revert to the basal level (Figure 2b), resulting in almost complete eradication of all three strains in the presence of 0.5 μg/ml ciprofloxacin (Figure 2a). To confirm whether the survival of E62 in the presence of ciprofloxacin was due to overexpression of biofilm genes, RT-PCR was performed. Compared to the level recorded in LB broth without antibiotic, the expression level of *pgaABCD* and *yjgK*, a gene regulating biofilm formation, was found to be upregulated by over 100-folds in the presence of 0.5 μg/ml ciprofloxacin, suggesting that high-level expression of biofilm formation genes was elicited in this strain and contributed to its survival in the presence of ciprofloxacin (Figure 2c). To test the relative degree of contribution of the *pgaA* and *hipA* gene product in the survival of strain E62 upon ciprofloxacin treatment, the minimum biofilm eliminating concentration (MBEC) of various test strains were determined as previously described.^18^ The MBEC of ciprofloxacin was determined to be 1 μg/ml for strain BW25113; in contrast, the corresponding values for the *pgaA* and *hipA* knockout strains were less than 0.03 and 0.25 μg/ml, respectively. On the other hand, the mutation prevention concentration (MPC) of ciprofloxacin of the test strains were also determined and found to be 0.008 μg/ml for strain BW25113, whereas those of the *pgaA* and *hipA* knockout strain were less than 0.002 μg/ml and 0.004 μg/ml, respectively, suggesting that both the *pgaA* and *hipA* genes played a role in the survival of *E. coli* under ciprofloxacin pressure. The data also suggest that the lower survival rate of E2 under ciprofloxacin selection pressure in the GI tract was due to the lack of *hipAB* genes, which was reported to play an important role in persister formation.^19^

### *Biofilm formation mediated by* pgaABD *gene cluster in* E. coli *strain E62 contribute to its better survival in rat GI tract*

To further confirm whether strain E62 was a more efficient survivor/persister in rat GI tract upon an antibiotic treatment, a 1:1 mixture of the *E. coli* BW25113 strain and the corresponding biofilm gene knockout mutant, BW25113::*ΔpgaA*, was introduced into the rat GI tract followed by ciprofloxacin treatment. Our *in vivo* data showed that the wild type BW25113 strain was much harder to be eradicated in the GI tract of rat even when the animals were treated with 5 mg/kg of ciprofloxacin when compared to BW25113::*ΔpgaA*, which disappeared upon treatment of only 0.05 mg/kg ciprofloxacin (Figure 2e, f). These data indicated that biofilm formation plays a crucial role in evolving into phenotypic resistance against ciprofloxacin.

To further confirm that *E. coli* harboring *pgaABCD* was present in the GI tract of rats as antibiotic-resistant bacterial progenitor cells, PCR assay targeting *pgaABCD* genes was used to assess the composition of the commensal *E. coli* population in five Sprague-Dawley (SD) rats before and after ciprofloxacin treatment, in order to confirm that the exceptionally high resistance formation potential exhibited by *pgaABCD*-bearing strains was a common phenomenon. Upon screening 500 *E. coli* strains recovered from five rats that did not harbor *E. coli* strains that exhibited reduced susceptibility to ciprofloxacin, we confirmed that only 3% of the strains tested carried *pgaABCD* before ciprofloxacin treatment. Upon treatment with ciprofloxacin for 24 h, however, the majority (99%) of the 500 commensal *E. coli* strains tested in these rats were found to harbor the *pgaABCD* genes (**Fig S3**).

### *A vast majority of clinical strains which harbored QRDR mutations contained the* pgaABCD *genes*

**Figure 4.**
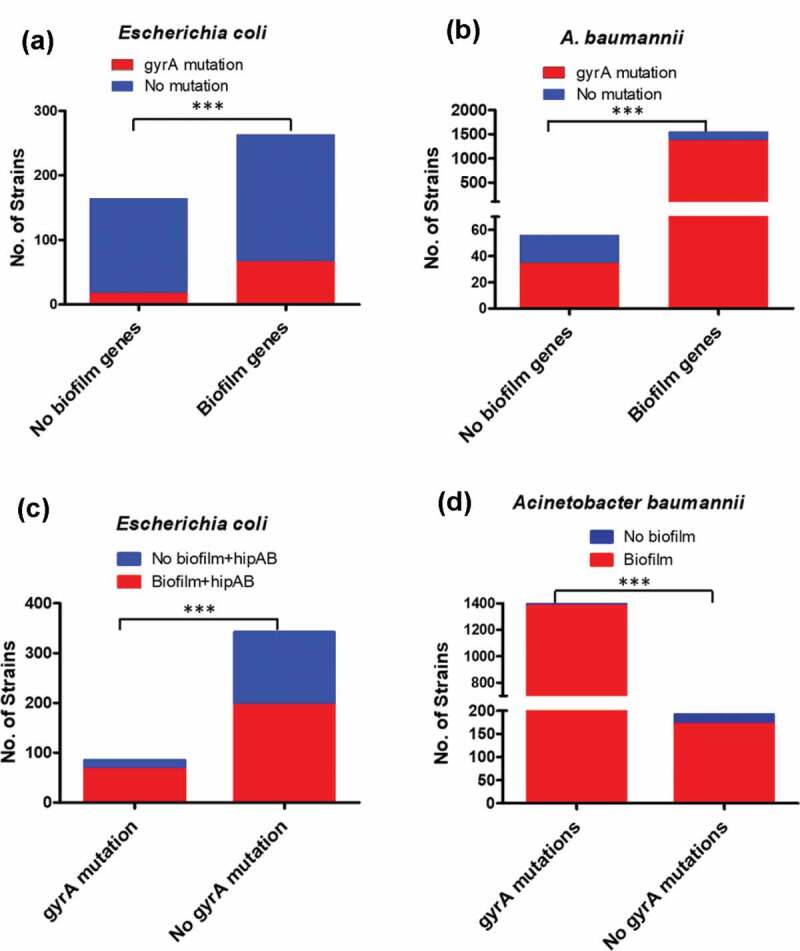
**Number and proportion of clinical strains with or without biofilm genes that harbored QRDR mutations**. (a) The number and proportion of clinical *E. coli* strains with or without biofilm genes that harbored mutations in the QRDR region of the *gyrA* gene; (b) The number and proportion of *A. baumannii* strains with or without biofilm genes that harbored mutations in the QRDR region of the *gyrA* gene. The number and proportion of clinical *E. coli* and *A. baumannii* strains with or without mutations in the QRDR region of the *gyrA* gene that harbored the biofilm genes are depicted in (c) and (d) respectively. Data were analyzed using two-tailed unpaired student’s *t* test using Prism Graphpad 7.0

As *de-novo* mutations that occur under clinical antibiotic selection pressure are a major route by which bacteria evolve to become antibiotic resistant, we investigated whether the majority of antibiotic-resistant clinical strains harboring target gene mutations possessed both the *pgaABCD* and *hipAB* genes observable in the E62 strain.^20^ In this work, the QRDR region of the *gyrA* gene of *E.coli*^21^ and *Acinetobacter baumannii*^22^ was selected as the query sequence, and BLASTP (Evalue = 1e-10) was used to identify the target mutations. Among the 164 *E. coli* strains that did not harbor the *pgaABCD* and *hipAB* genes, QRDR mutations were detected in 18 (11%) strains. However, among the 263 *E. coli* strains which harbored the *pgaABCD* and *hipAB* genes, QRDR mutations were detected in 67 (25%) strains. In other words, among a total of 85 strains that harbored QRDR mutations, as much as 79% (67 strains) contained the *pgaABCD* and *hipAB* genes (Figure 4). A similar phenomenon was also found in *A. baumannii*, in which only 56 genomes without the *pgaABCD* genes were found, among which a total of 35 genomes were found to harbor QRDR target mutations (63%); on the other hand, a total of 1552 genomes, which possessed the *pgaABCD* genes were recorded in the Genbank, among which as many as 1385 were found to harbor QRDR mutations (89%). These data, therefore, indicated that as much as 98% of *A. baumannii* strains that harbor QRDR mutations (1385 out of 1420 strains) possess the *pgaABCD* genes, while only 67% (35/56) of *A. baumannii* strains carried QRDR mutations (Figure 4). Taken together, our data suggested that the proportion of QRDR mutations in *E. coli* and *A. baumannii* that carried *the pgaABCD* gene cluster was significantly higher (*P* < .0001) than those that did not carry this gene cluster, suggesting the close association of *pgaABCD* gene cluster and QRDR mutations.

### Discussion

It should be noted that the genetic complexity of the gut microbiome could not be elucidated previously due to a lack of efficient whole genome and metagenomic sequencing techniques, which can reveal details of genetic changes during resistance development in a bacterial population. Our works show that among the wide range of detectable genetic differences between the prevalent microbiome strains (specific to *E. coli* in this study) and the rare progenitor strains which could evolve into resistant organisms under antibiotic selection pressure, the *pgaABCD* genes, which are responsible for translocation of the biofilm adhesion molecule de-N-acetylated poly-β-1,6-N-acetyl-d-glucosamine (dPNAG) across the outer membrane, play a key role in conferring the ability of the progenitor strains to survive against the detrimental effects of antibiotic, without which mutational changes leading to expression of resistance phenotypes would not be possible. It is known that bacteria residing within the biofilm are 100–1000 times more tolerant to antibiotics than the planktonic organisms.^16^ Synthesis of protective, biofilm-like structures may render bacteria in the gut microbiome physically tolerant to antibiotics, thereby enhancing the survival fitness and hence the chance of evolving into resistant organisms during the process of antimicrobial treatment. Consistently, genome database mining and analysis demonstrated that the vast majority of fluoroquinolone-resistant clinical strains which harbored QRDR mutations were biofilm-forming organisms. Such a finding is highly significant considering the fact that biofilm-forming strains normally constitute a small proportion of the gut microbiome population. We would also like to point out that the survival of antimicrobial resistance progenitor cells in the gut microbiome is different from bacterial persisters, which shifts to a slow growth rate and survive upon antibiotic treatment. However, these persister cells remain antibiotic susceptible. Although the toxin-antitoxin system, HipAB, is involved in both processes, antibiotic resistance progenitor is different from bacterial persister cells.

Based on their genetic and phenotypic characteristics, we herein coin the term ‘bacterial resistance progenitor cells’ to describe a subpopulation of *E. coli* in gut microbiome strains in animals that exhibit the extraordinary potential to evolve into resistant organisms upon treatment with fluoroquinolone antibiotics. We speculate that such strains might commonly exist in other bacterial species in human and different animals entails further investigation. Progenitors for other antibiotics might also exist in the gut microbiome of human and animals. We expect that the proportion of resistance progenitor cells in the gut microbiome, as well as their genetic composition, varies wildly among the human and animal population, depending on the history of antibiotic consumption and other factors. Nevertheless, we believe that eradication of such strains from the human/animal gut microbiome represents a novel and effective strategy to drastically reduce the rate of drug-resistant hospital infections through interrupting the evolution and transmission of resistant organisms. This is a novel antibiotic resistance-control concept in which the breeding and storage sites of resistant strains are simultaneously targeted for the first time to reduce the chance of resistance development, minimize contamination of the environment by resistant organisms, and reduce the rate of drug-resistant opportunistic infections. This strategy is likely to be successful, at least for organisms in the family of *Enterobacteriaceae* which originate from the gastrointestinal tract, because members of *Enterobacteriaceae* are known to exhibit a high resistance rate and are the major group of organisms responsible for causing drug-resistant hospital/opportunistic, community-based infections.

In conclusion, this study for the first time dissected the role of subspecies of *E. coli* on ciprofloxacin resistance formation in gut microbiome upon antibiotic treatment. Our findings suggested that genes encoding biofilm-forming ability played an important role in conferring gut *E. coli* the ability to evolve into resistant strains upon a prolonged antibiotic treatment, and that such strains therefore could be considered as antibiotic resistance progenitor cells in the gut microbiome. This study provided important insight into the development of new strategies to control the emergence and evolution of multidrug-resistant bacteria in the human and animal microbiome.

## Materials and methods

### Bacterial strains and culture condition

Representative strains were cultured and collected from MacConkey (MAC) agar plates before and after antibiotic treatment, and identified by MALDI-TOF MS. *E. coli* isolates recovered before and after treatment were subjected to pulsed-field gel electrophoresis (PFGE) upon Xbal digestion as previously described.^7^ All *E. coli* strains were cultured in Luria-Bertani (LB) broth or agar at 37 C except for specific nutritional assays. For *in vitro* nutritional competition experiments, *E. coli* strains were first cultured in MOPS medium (1.32 mM K_2_HPO_4_, 9.52 mM NH_4_Cl, 0.523 mM MgCl_2_, 0.276 mM K_2_SO_4_) aerobically overnight before the experiments.^23^ Rats were purchased from the Guangdong Medical Laboratory Animal Center and allowed free access to food and water. All SD rats used in this study were subjected to a screening of quinolone-resistant *E. coli* in the GI tract and only rats that did not harbor quinolone-resistant *E. coli* were used in this study.

### Induction of antimicrobial resistance development in the GI tract of rats

Experiments designed to test the effect of antimicrobial treatment on the gut microbiome of animals were performed as previously described with slight modification.^7^ Three individually housed SD rats were subjected to an oral antibiotic treatment scheme, which involved three consecutive rounds of antibiotic treatment and withdrawal sessions. Briefly, all three rats were first treated with 1 mg/kg body weight of ciprofloxacin through gavage for 5 days, followed by withdrawal of antibiotic for 4 days; the second round of ciprofloxacin retreatment was then initiated and lasted for 5 days, followed by a cessation period of 4 days, after which the third round of ciprofloxacin retreatment resumed and lasted for another 5 days, followed by 3 days of monitoring without antibiotic treatment. At indicated time points, fresh feces (50–100 mg) were collected and re-suspended in 1 ml of saline. The suspension was mixed and diluted 10-fold in saline. Up to 100 μl of suspension were plated on MacConkey agar containing 0 or 0.25 mg/L of ciprofloxacin, followed by incubation at 37 C for 12 h and enumeration of the colony-forming units (CFUs) per gram of feces. Colonies that exhibited typical morphology of *E. coli* on MacConkey plate (pink to rose-red, large regular colonies) were picked and subjected to confirmation of species identity by MALDI-TOF.

### *Whole genome sequencing and bioinformatics analysis of representative* E. coli *strains*

Genomic DNA of representative *E. coli* isolates was extracted using the Invitrogen PureLink genomic DNA mini kit, following the manufacturer’s instructions. Genomic libraries were prepared using the NEBNext® Ultra™ II DNA Library Prep Kit for Illumina®(NEB) to obtain 2 × 150 bp paired-end reads for NextSeq 500 Illumina sequencing. After *de novo* assembly by SOAPdenovo2,^24^ the contigs were uploaded to CSI Phylogeny 1.2 (https://cge.cbs.dtu.dk/services/CSIPhylogeny/) for phylogenetic analysis. *E. coli* strain K-12 strain MG1655(NC_000913.3) was chosen as the reference genome.^25^ The SNP profile was obtained using iTOL to construct the Phylogenetic tree (http://itol.embl.de/index.shtml).^26^ To obtain the completed genome of strains E3 and E62, PacBio RSII single-molecule, real-time (SMRT) sequencing was performed at the Wuhan Institute of Biotechnology, Wuhan, China to create libraries of 20 kb. SPAdes was utilized to perform hybrid-assembly to obtain the complete sequence of these two isolates.^27^ The subsystem categories of the predicted ORFs were obtained from the SEED viewer.^28^ The complete genome sequences of the *E. coli* strains in this study were as follows: *E. coli* E62 (CP022393), *E. coli* E2 (NJIS00000000), *E. coli* E3 (NJIR00000000), *E. coli* E15 (NJIQ00000000), *E. coli* E21(NJIP00000000), and *E. coli* E27 (NJIO00000000).

### Gene knockout mutants

For functional characterization of a specific gene, BW25113 and the corresponding gene knockout mutants were used. All gene knockout mutants were obtained from Keio Collection (CGSC) and subjected to confirmation by PCR.

### Assays to determine mutation prevention concentration and minimum biofilm eliminating concentration

Mutation prevention concentration (MPC) of selected *E. coli* strains was determined as previously described.^29^ The assay for minimum biofilm eliminating concentration (MBEC) was performed as previously described.^30^

### Assays for biofilm formation and biofilm-related gene expression

Biofilm assay was conducted in 96-well polystyrene plates containing LB broth. Bacteria were inoculated into the wells and subjected to incubation at 37°C for 24 h without shaking. The cell density (turbidity at OD_595_) of the bacterial culture and formation of biofilm (absorbance at OD_540_) in the wells were measured upon staining with 0.1% crystal violet. Biofilm formation potential was determined by normalizing the total amount of detectable biofilm with the degree of bacterial growth of each culture. The proton pump inhibitor (PPI) esomeprazole^17^ was used as an inhibitor of biofilm formation and was added to produce a final concentration of 32 μg/ml in an attempt to test the effect of biofilm formation on bacterial survival. The expression status of biofilm genes *pgaABC* and *ygjK* was determined. The *ygjK* gene is known to encode a glucosidase,^9^ which serves as a biofilm modulator.^10^ Briefly, the expression level of the biofilm genes was determined with and without ciprofloxacin selection pressure. Upon treatment with ciprofloxacin, total RNA was extracted using the RNeasy kit (QIAGEN) and treated with the Turbo DNA free kit (Ambion, Austin, TX) following the manufacturer’s instructions. Reverse transcription analysis was performed using the SuperScript III quantitative one-step kit (Invitrogen), with 16s rRNA being used to normalize the expression level of the test genes so that the expression levels of biofilm genes recorded under different test conditions could be compared. The 16s rRNA primers and biofilm gene primers were used as previously described.^10,31^ SYBR Green master mix (Applied Biosystems) was used to perform Real-time PCR. Results were analyzed by the iQ5 optical system software.

### In vivo *assays*

To test the survival of *E. coli* and the corresponding biofilm knockout mutants in rat GI tract upon treatment with ciprofloxacin, twenty-eight male SD rats were treated with streptomycin as described above; a 1:1 mixture of wild type *E. coli* strain (BW25113) and biofilm formation locus knockout strains (Δ*pgaA*) were added into the drinking water and fed to the rats for 12 hours. To confirm the selection of *E. coli* carrying biofilm formation genes *pgaABC*, SD rats were subjected to a screening of *pgaABC*-bearing *E. coli* before and after ciprofloxacin treatment. All animals were then treated with ciprofloxacin (0, 0.05, 0.5, and 5 mg/kg) for 24 hours. The experimental procedures have been approved by the Research Animal Care and Use Committee of the Hong Kong Polytechnic University.

### *Comparison of the genetic characteristics of* E. coli *strains in the NCBI database*

To check for the presence of *gyrA* mutations and *pgaABCD* genes in clinical *E. coli* isolates, a total of 427 clinical *E. coli* genome sequences and 1608 *A. baumannii* genomes were downloaded from the NCBI genome database (https://www.ncbi.nlm.nih.gov/genome/) in fasta format and analyzed using the pipeline developed in our laboratory. Detailed information regarding the source of the isolates was obtained from Pathogen Detection BETA(http://www.ncbi.nlm.nih.gov/pathogens/). Bacterial sequences were annotated by prokka.^32,33^ Core and pan genes were analyzed by Roary.^34^ The QRDR region of the *gyrA* gene of *E.coli*^21^ and *Acinetobacter baumannii*^22^ was selected as the query sequence, and BLASTP (Evalue = 1e-10) was used to identify the target mutations.

## Supplementary Material

Supplemental Material

## Data Availability

All data generated or analyzed during this study are included in this published article and its supplementary information files. All supporting data are available upon request or included in the supplementary materials.

## References

[1] Kumarasamy KK, Toleman MA, Walsh TR, Bagaria J, Butt F, Balakrishnan R, Chaudhary U, Doumith M, Giske CG, Irfan S, *et al*. Emergence of a new antibiotic resistance mechanism in India, Pakistan, and the UK: a molecular, biological, and epidemiological study. Lancet Infect Dis. 2010;10(9):597–602. doi:10.1016/S1473-3099(10)70143-2. 20705517 PMC2933358

[2] Nordmann P, Dortet L, Poirel L. Carbapenem resistance in Enterobacteriaceae: here is the storm! Trends Mol Med. 2012;18(5):263–272. doi:10.1016/j.molmed.2012.03.003. 22480775

[3] Rochford C, Sridhar D, Woods N, Saleh Z, Hartenstein L, Ahlawat H, Whiting E, Dybul M, Cars O, Goosby E, *et al*. Global governance of antimicrobial resistance. Lancet. 2018;391(10134):1976–1978. doi:10.1016/S0140-6736(18)31117-6. 29864011

[4] Lee SM, Donaldson GP, Mikulski Z, Boyajian S, Ley K, Mazmanian SK. Bacterial colonization factors control specificity and stability of the gut microbiota. Nature. 2013;501(7467):426–429. doi:10.1038/nature12447. 23955152 PMC3893107

[5] Faith JJ. The long-term stability of the human gut microbiota. science. 2013;341:1237439. doi:10.1126/science.1237439. 23828941 PMC3791589

[6] Wang X, Preston JF 3rd, Romeo T. The pgaABCD locus of Escherichia coli promotes the synthesis of a polysaccharide adhesin required for biofilm formation. J Bacteriol. 2004;186:2724–2734. doi:10.1128/jb.186.9.2724-2734.2004. 15090514 PMC387819

[7] Lin D, Chen K, Li R, Liu L, Guo J, Yao W, Chen S. Selection of target mutation in rat gastrointestinal tract E. coli by minute dosage of enrofloxacin. Front Microbiol. 2014;5:468. doi:10.3389/fmicb.2014.00468. 25237308 PMC4154546

[8] Alikhan N-F, Petty NK, Zakour NLB, Beatson SA. BLAST Ring Image Generator (BRIG): simple prokaryote genome comparisons. BMC Genomics. 2011;12(1):1. doi:10.1186/1471-2164-12-402. PMC3163573 21824423

[9] Kurakata Y. Structural insights into the substrate specificity and function of Escherichia coli K12 YgjK, a glucosidase belonging to the glycoside hydrolase family 63. J Mol Biol. 2008;381:116–128. doi:10.1016/j.jmb.2008.05.061. 18586271

[10] Kim Y, Wang X, Ma Q, Zhang X-S, Wood TK. Toxin-antitoxin systems in Escherichia coli influence biofilm formation through YjgK (TabA) and fimbriae. J Bacteriol. 2009;191:1258–1267. 19060153 10.1128/JB.01465-08 PMC2632003

[11] Shrestha R, Khanal S, Poudel P, Khadayat K, Ghaju S, Bhandari A, Lekhak S, Pant ND, Sharma M, Marasini BP, *et al*. Extended spectrum beta-lactamase producing uropathogenic Escherichia coli and the correlation of biofilm with antibiotics resistance in Nepal. Ann Clin Microbiol Antimicrob. 2019;18(1):42. doi:10.1186/s12941-019-0340-y. 31847837 PMC6918583

[12] Chen K-M, Chiang M-K, Wang M, Ho H-C, Lu M-C, Lai Y-C. The role of pgaC in Klebsiella pneumoniae virulence and biofilm formation. Microb Pathog. 2014;77:89–99. doi:10.1016/j.micpath.2014.11.005. 25450884

[13] Itoh Y, Rice JD, Goller C, Pannuri A, Taylor J, Meisner J, Beveridge TJ, Preston JF, Romeo T. Roles of pgaABCD genes in synthesis, modification, and export of the Escherichia coli biofilm adhesin poly-beta-1,6-N-acetyl-D-glucosamine. J Bacteriol. 2008;190(10):3670–3680. doi:10.1128/JB.01920-07. 18359807 PMC2394981

[14] Nguyen D, Joshi-Datar A, Lepine F, Bauerle E, Olakanmi O, Beer K, McKay G, Siehnel R, Schafhauser J, Wang Y. Active starvation responses mediate antibiotic tolerance in biofilms and nutrient-limited bacteria. science. 2011;334(6058):982–986. doi:10.1126/science.1211037. 22096200 PMC4046891

[15] Lewis K. Persister cells. Annu Rev Microbiol. 2010;64(1):357–372. doi:10.1146/annurev.micro.112408.134306. 20528688

[16] Olsen I. Biofilm-specific antibiotic tolerance and resistance. Eur J Clin Microbiol Infect Dis. 2015;34:877–886. 25630538 10.1007/s10096-015-2323-z

[17] Singh V, Arora V, Alam MJ, Garey KW. Inhibition of biofilm formation by esomeprazole in Pseudomonas aeruginosa and Staphylococcus aureus. Antimicrob Agents Chemother. 2012;56(8):4360–4364. doi:10.1128/AAC.00544-12. 22664967 PMC3421605

[18] Sepandj F, Ceri H, Gibb A, Read R, Olson M. Minimum inhibitory concentration (MIC) versus minimum biofilm eliminating concentration (MBEC) in evaluation of antibiotic sensitivity of gram-negative bacilli causing peritonitis. Peritoneal Dialysis Int. 2004;24:65–67. 15104338

[19] Keren I, Shah D, Spoering A, Kaldalu N, Lewis K. Specialized persister cells and the mechanism of multidrug tolerance in Escherichia coli. J Bacteriol. 2004;186(24):8172–8180. doi:10.1128/JB.186.24.8172-8180.2004. 15576765 PMC532439

[20] Wellington EM, Boxall AB, Cross P, Feil EJ, Gaze WH, Hawkey PM, Johnson-Rollings AS, Jones DL, Lee NM, Otten W, *et al*. The role of the natural environment in the emergence of antibiotic resistance in Gram-negative bacteria. Lancet Infect Dis. 2013;13(2):155–165. doi:10.1016/S1473-3099(12)70317-1. 23347633

[21] Nakamura S. Mechanisms of quinolone resistance. J Infec Chemother. 1997;3(3):128–138. doi:10.1007/BF02491502.

[22] Vila J, Ruiz J, Goni P, Marcos A, De Anta TJ. Mutation in the gyrA gene of quinolone-resistant clinical isolates of Acinetobacter baumannii. Antimicrob Agents Chemother. 1995;39(5):1201–1203. doi:10.1128/AAC.39.5.1201. 7625818 PMC162713

[23] Neidhardt FC, Bloch PL, Smith DF. Culture medium for enterobacteria. J Bacteriol. 1974;119(3):736–747. doi:10.1128/JB.119.3.736-747.1974. 4604283 PMC245675

[24] Luo R, Liu B, Xie Y, Li Z, Huang W, Yuan J, He G, Chen Y, Pan Q, Liu Y, *et al*. SOAPdenovo2: an empirically improved memory-efficient short-read de novo assembler. GigaScience. 2012;1(1):1. doi:10.1186/2047-217X-1-18. 23587118 PMC3626529

[25] Kaas RS, Leekitcharoenphon P, Aarestrup FM, Lund O. Solving the problem of comparing whole bacterial genomes across different sequencing platforms. PLoS One. 2014;9:e104984. 25110940 10.1371/journal.pone.0104984 PMC4128722

[26] Letunic I, Bork P. Interactive tree of life (iTOL) v3: an online tool for the display and annotation of phylogenetic and other trees. Nucleic Acids Res. 2016;44:W242–5. doi:10.1093/nar/gkw290 27095192 PMC4987883

[27] Nurk S, Bankevich A, Antipov D, Gurevich A, Korobeynikov A, Lapidus A, Prjibelsky A, Pyshkin A, Sirotkin A, Sirotkin Y, et al. Assembling Genomes and Mini-metagenomes from Highly Chimeric Reads. In: Deng M., Jiang R., Sun F., Zhang X, editors. Research in Computational Molecular Biology. RECOMB 2013. Lecture Notes in Computer Science, vol 7821. Berlin, Heidelberg: Springer; 2013. 10.1007/978-3-642-37195-0_13

[28] Overbeek R, Olson R, Pusch GD, Olsen GJ, Davis JJ, Disz T, Edwards RA, Gerdes S, Parrello B, Shukla M, *et al*. The SEED and the Rapid Annotation of microbial genomes using Subsystems Technology (RAST). Nucleic Acids Res. 2014;42:D206–D214. 24293654 10.1093/nar/gkt1226 PMC3965101

[29] Marcusson LL, Olofsson SK, Komp Lindgren P, Cars O, Hughes D. Mutant prevention concentrations of ciprofloxacin for urinary tract infection isolates of Escherichia coli. J Antimicrob Chemother. 2005;55(6):938–943. doi:10.1093/jac/dki136. 15860549

[30] Naparstek L, Carmeli Y, Navon-Venezia S, Banin E. Biofilm formation and susceptibility to gentamicin and colistin of extremely drug-resistant KPC-producing Klebsiella pneumoniae. J Antimicrob Chemother. 2014;69(4):1027–1034. doi:10.1093/jac/dkt487. 24408988

[31] Clifford RJ, Milillo M, Prestwood J, Quintero R, Zurawski DV, Kwak YI, Waterman PE, Lesho EP, Mc Gann P. Detection of bacterial 16S rRNA and identification of four clinically important bacteria by real-time PCR. PloS One. 2012;7(11):e48558. doi:10.1371/journal.pone.0048558. 23139793 PMC3490953

[32] Kanehisa M, Sato Y, Morishima MK. BlastKOALA and GhostKOALA: KEGG tools for functional characterization of genome and metagenome sequences. J Mol Biol. 2016;428(4):726–731. doi:10.1016/j.jmb.2015.11.006. 26585406

[33] Seemann T. Prokka: rapid prokaryotic genome annotation. Bioinformatics. 2014;30:btu153. 10.1093/bioinformatics/btu153 24642063

[34] Page AJ, Cummins CA, Hunt M, Wong VK, Reuter S, Holden MTG, Fookes M, Falush D, Keane JA, Parkhill J, *et al*. Roary: rapid large-scale prokaryote pan genome analysis. Bioinformatics. 2015;31(22):3691–3693. doi:10.1093/bioinformatics/btv421. 26198102 PMC4817141

