# Supplementary material for: Contribution of biofilm formation genetic locus, pgaABCD, to antibiotic resistance development in gut microbiome
Source: Gut Microbes. 2020 Nov 16;12(1):1842992. doi: 10.1080/19490976.2020.1842992 (PMC7671071; doi:10.1080/19490976.2020.1842992)
Supplement: Supplemental Material [file KGMI_A_1842992_SM2438.docx]

**Supplementary Materials**

**Contribution of biofilm formation genetic locus, *pgaABCD*, to antibiotic resistance development in gut microbiome**

Dachuan Lin^1,2^, Kaichao Chen^3^, Jiubiao Guo^1,2^, Lianwei Ye^3^, Ruichao Li^4^, Edward Wai Chi Chan^2^, Sheng Chen^3*^

Running title: Resistance development in gut microbiome

^1^Guangdong Provincial Key Laboratory of Regional Immunity and Disease, Department of Pathology Biology, School of Medicine, Shenzhen University, Shenzhen, China

^2^State Key Laboratory of Chemical Biology and Drug Discovery, Department of Applied Biology and Chemical Technology, The Hong Kong Polytechnic University, Hung Hom, Kowloon, Hong Kong.

^3^Department of Infectious Diseases and Public Health, Jockey Club College of Veterinary Medicine and Life Sciences, City University of Hong Kong, Kowloon, Hong Kong

^4^Jiangsu Co-Innovation Center for Prevention and Control of Important Animal Infectious Diseases and Zoonoses, College of Veterinary Medicine, Yangzhou University, Yangzhou, People's Republic of China

*Corresponding authors: Sheng Chen, Tel: (852)-3442-5782; Email: [shechen@cityu.edu.hk](mailto:shechen@cityu.edu.hk);

**Supplementary Figures**


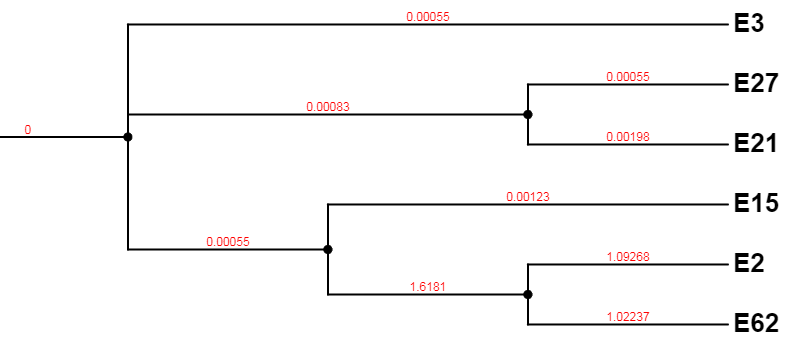


**Supplementary Figure S1. Phylogenetic tree of six representative *E. coli* strains.** *After de novoa ssembly, contigs were* uploaded to CSI Phylogeny 1.2 (<https://cge.cbs.dtu.dk/services/CSIPhylogeny/>). *Escherichia coli* strain K-12 substr. MG1655(NC_000913.3) was chosen as the reference genome (Kaas et al., 2014). The iTOL software was used to create the Phylogenetic tree based on the SNP profiles of the test strains (http://itol.embl.de/index.shtml)(Letunic and Bork, 2016). E2 and E62 showed closest genetic relationship, while others exhibited close genetic relationship to each other, while distal to E2 and E62. The trees are drawn with ignored the scale. The red number (next to the branch) indicated the branch lengths measured in the number of substitutions per site.

#
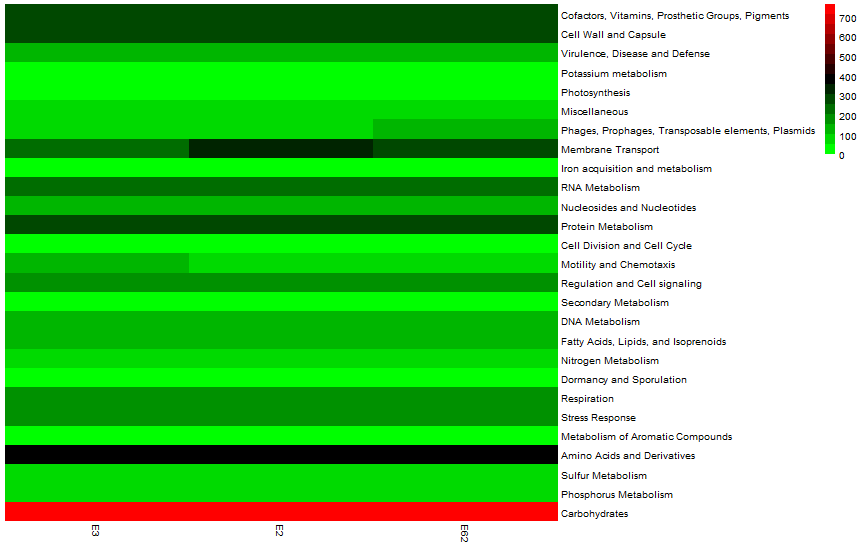


**Supplementary Figure S2. Kegg category distributions of predicted ORFs.** The heat map indicated the number of genes in each category according to Kegg annotation. A total of 50 genes uniquely harboured by strain E3 were identified, 21 of which were phage and plasmid replicate genes, one was involved in synthesis of folate, which is beneficial to the host, one for L-rhamnonate metabolism (although strain E62 grew better in this substrate), three were responsible for synthesis of cell wall and capsule, six were involved in metabolism of D-allose, which is extremely rare in nature(Yamaguchi et al., 2008), one was involved in antibiotic synthesis, five were hypothetical proteins. The rest were genes involved in serine metabolism. The detail data can be seen from supplementary dataset 1.


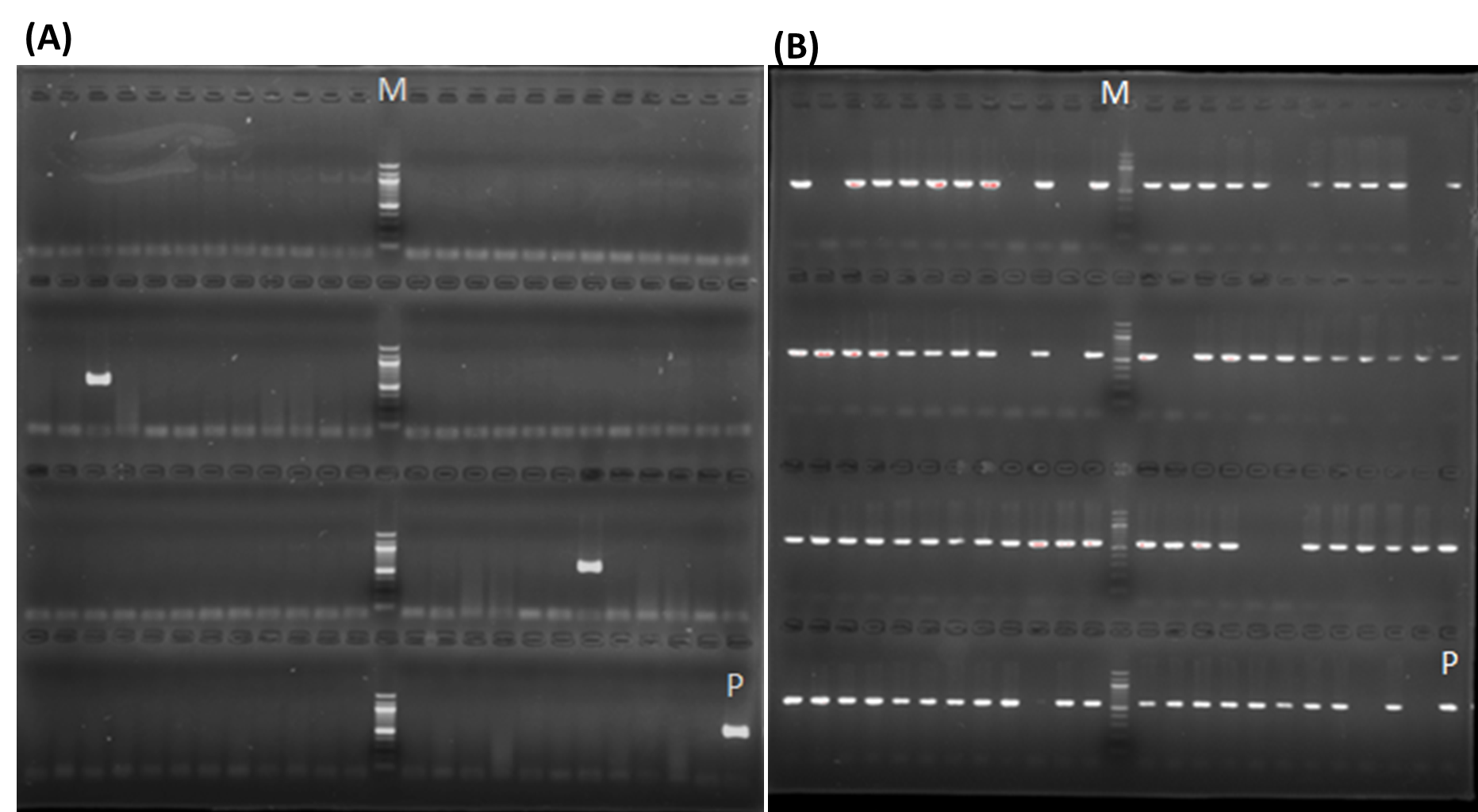


**Supplementary Figure S3. Prevalence of commensal E. coli that carried *pgaA* before and after treatment with ciprofloxacin.** Each of 500 E. coli strains recovered from five rats which did not harbor E. coli strains that exhibited reduced susceptibility to ciprofloxacin, before and after ciprofloxacin treatment for 24h. These E. coli strains were subjected to PCR screening of the presence of *pgaA* as previously described before(Subashchandrabose et al., 2013). Our data showed that around 3% of the strains tested carried pgaABCD before ciprofloxacin treatment, while the majority (99%) of the 500 commensal E. coli strains tested in these rats were found to harbor the pgaABCD genes after ciprofloxacin treatment. A representative gel of PCR screening of E. coli before ciprofloxacin treatment (A) and after cirpfloxacin treatment (B) were shown. P: Positive control.

**Supplementary references:**

Kaas, R.S., Leekitcharoenphon, P., Aarestrup, F.M., and Lund, O. (2014). Solving the problem of comparing whole bacterial genomes across different sequencing platforms. PLoS One *9*, e104984.

Letunic, I., and Bork, P. (2016). Interactive tree of life (iTOL) v3: an online tool for the display and annotation of phylogenetic and other trees. Nucleic acids research, gkw290.

Subashchandrabose, S., Smith, S., Spurbeck, R., Kole, M., and Mobley, H. (2013). Genome-wide detection of fitness genes in uropathogenic Escherichia coli during systemic infection. PLoS pathogens *9*, e1003788.

Yamaguchi, F., Takata, M., Kamitori, K., Nonaka, M., Dong, Y., Sui, L., and Tokuda, M. (2008). Rare sugar D-allose induces specific up-regulation of TXNIP and subsequent G1 cell cycle arrest in hepatocellular carcinoma cells by stabilization of p27kip1. International journal of oncology *32*, 377-386.
